# A novel strategy for optimal component formula of anti-PRRSV from natural compounds using tandem mass tag labeled proteomic analyses

**DOI:** 10.1186/s12917-022-03184-w

**Published:** 2022-05-14

**Authors:** Hua Zhang, Zhigang Cao, Panpan Sun, Ajab Khan, Jianhua Guo, Yaogui Sun, Xiuju Yu, Kuohai Fan, Wei Yin, E Li, Na Sun, Hongquan Li

**Affiliations:** 1 Shanxi key lab. for modernization of TCVM, College of Veterinary Medicine, Shanxi Agricultural University, Shanxi 030801 Taigu, China; 2 Laboratory Animal Center, Shanxi Agricultural University, Shanxi 030801 Taigu, China; 3 Department of Veterinary Pathobiology, Schubot Exotic Bird Health Center, Texas A&M University, TX 77843 College Station, USA; 4 Haowei Biotechnology Co., Ltd, Tianjin, 300000 China

**Keywords:** PRRSV, Matrine, Glycyrrhizic acid, Tea saponin, TMT, IFN-β

## Abstract

**Background:**

Porcine Reproductive and Respiratory Syndrome (PRRS) is one of the most important porcine viral diseases which have been threatening the pig industry in China. At present, most commercial vaccines fail to provide complete protection because of highly genetic diversity of PRRSV strains. This study aimed to optimize a component formula from traditional Chinese medicine(TCM)compounds with defined chemical characteristics and clear mechanism of action against PRRSV.

**Methods:**

A total of 13 natural compounds were screened for the anti-PRRSV activity using porcine alveolar macrophages (PAMs). Three compounds with strong anti-PRRSV activity were selected to identify their potential protein targets by proteomic analysis. The optimal compound formula was determined by orthogonal design based on the results of proteomics. MTT assay was used to determine the maximum non-cytotoxic concentration (MNTC) of each compound using PAMs. QPCR and western blot were used to investigate the PRRSV N gene and protein expression, respectively. The Tandem Mass Tag (TMT) technique of relative quantitative proteomics was used to detect the differential protein expression of PAMs treated with PRRSV, matrine (MT), glycyrrhizic acid (GA) and tea saponin (TS), respectively. The three concentrations of these compounds with anti-PRRSV activity were used for orthogonal design. Four formulas with high safety were screened by MTT assay and their anti-PRRSV effects were evaluated.

**Results:**

MT, GA and TS inhibited PRRSV replication in a dose-dependent manner. CCL8, IFIT3, IFIH1 and ISG15 were the top four proteins in expression level change in cells treated with MT, GA or TS. The relative expression of IFIT3, IFIH1, ISG15 and IFN-β mRNAs were consistent with the results of proteomics. The component formula (0.4 mg/mL MT + 0.25 mg/mL GA + 1.95 μg/mL TS) showed synergistic anti-PRRSV effect.

**Conclusions:**

The component formula possessed anti-PRRSV activity in vitro, in which the optimal dosage on PAMs was 0.4 mg/mL MT + 0.25 mg/mL GA + 1.95 μg/mL TS. Compatibility of the formula was superposition of the same target with GA and TS, while different targets of MT. IFN-β may be one of the targets of the component formula possessed anti-PRRSV activity.

**Supplementary Information:**

The online version contains supplementary material available at 10.1186/s12917-022-03184-w.

## Background

Globally, porcine reproductive and respiratory syndrome (PRRS) is a viral swine disease causing huge economic losses to the swine industry. The causative agent, PRRS virus (PRRSV), is a positive stranded RNA virus approximately 15 kilobase (kb) in length. The major threat to PRRSV infection is persistent infection, secondary infection and co-infection [1]. The median total loss attributable to PRRS in Germany was 255 Euro per sow per year [2]. In China, a growing number of endemic strains have been emerged because of high genetic diversity and various RNA recombination patterns [3–5], and which is reason that the existing commercial vaccines could not provide enough protection. Therefore, the novel effective antiviral strategies are urgently needed to control the spread of PRRSV or at least mitigate the pathological damage to swine organs.

Traditional Chinese Medicines (TCM) have been widely used to treat various diseases and served as a source of inspiration for drug development [6]. Artemisinin, arsenic trioxide, berberine and Danshen are all successful demonstration of TCM contribution to the modern medicine [7]. TCM has been used as the antiviral drugs in the history, such as hepatitis B virus (HBV) [8], herpes simplex virus type 1 (HSV-1) [9], influenza virus A (H1N1) [10] and COVID-19 [11]. “Fei Yan No.1”, a TCM formula consisted with 13 traditional Chinese medicines, has been recommended by the Hubei Government for the prevention and control of COVID-19 [12]. In veterinary domain, tomatidine has been demonstrated to inhibit porcine epidemic diarrhea virus replication by targeting 3CL protease [13]. Some active compounds could inhibit PRRSV infection at a certain stage of the virus life cycle or improve the host immune system [14]. So far, most studies have been focusing on antiviral effect of natural compounds in vitro. TCM formulas contain numerus of medical materials relying on each other to achieve the therapeutic effect, it is impossible to clearly analyze the active ingredients quantitatively and qualitatively. The study of underlying mechanisms of TCM responsible for antiviral activity is still at infant stage.

The component formula with clear components and targeting multiple networks will overcome the limitations mentioned above. In this study, anti-PRRSV activities of 13 natural compounds possessing antiviral, anti-inflammatory activities were assessed using PRRSV infected porcine alveolar macrophages (PAMs, the target cells of PRRSV) model. The potential targets of each compound were analyzed with proteomic approaches (Tandem Mass Tag Labeling). The optimal combination of compounds demonstrated its anti-PRRSV activity through multiple targets was investigated by orthogonal design.

## Results

### Glycyrrhizic acid, Matrine and tea saponin possessed the anti-PRRSV activity

**Fig. 1:**
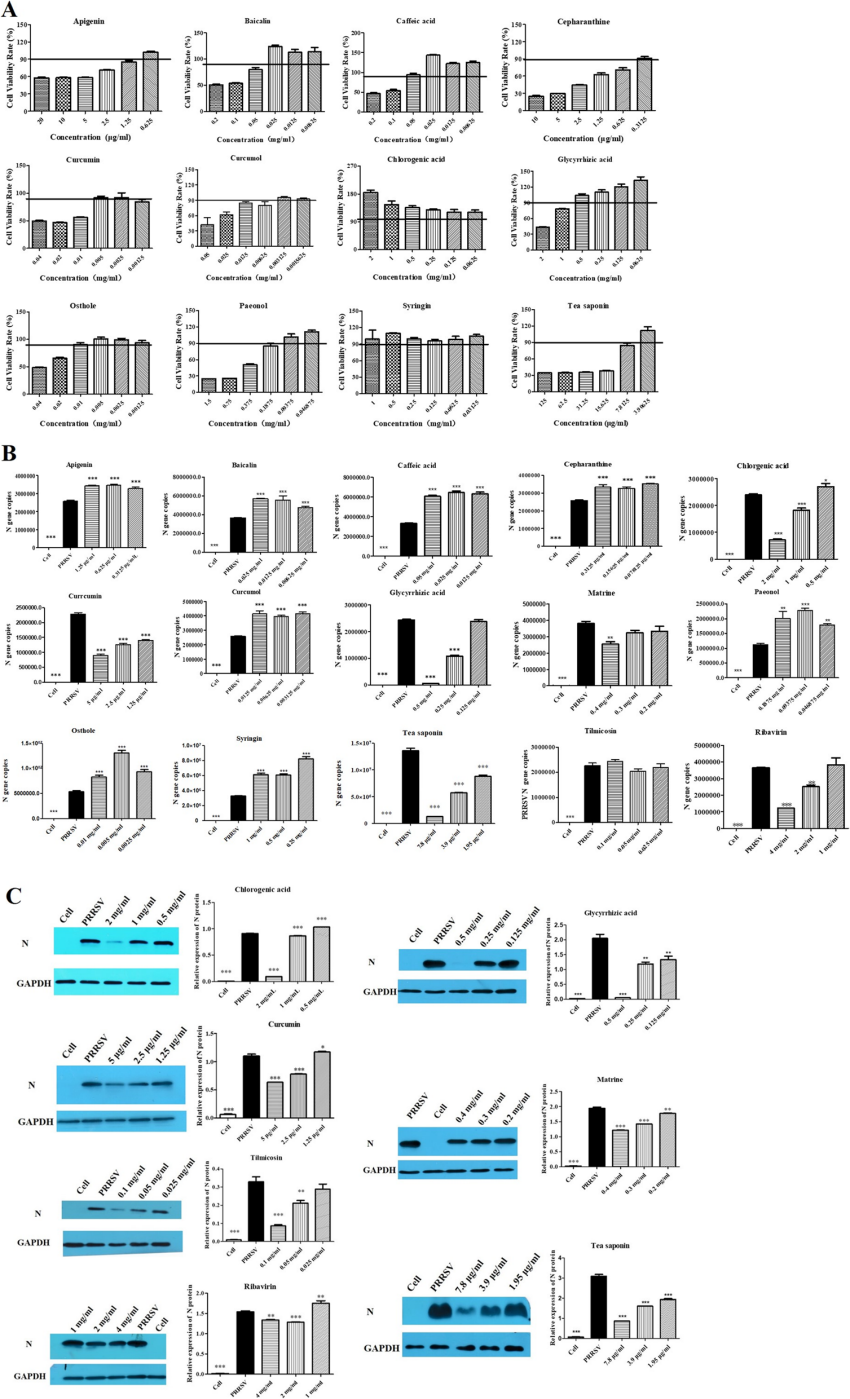
Cytotoxicity (**A)** and anti-PRRSV activity (**B** and **C**) of the screened compounds on PAMs. **A**: The maximum non-cytotoxic concentrations of the screened compounds was determined by MTT assay for the cell viability. **B** and **C**: The detection of PRRSV N gene (**B**) and protein (**C**) expression by qPCR and western blot. Cropped bolts are displayed. Compared with PRRSV group, the differences in gene and protein were significant, * means *p* < 0.05, ** means *p* < 0.01, *** means *p* < 0.001

The MNTC of the selected compounds were determined by MTT assay. As shown in Fig. 1A, both chlorogenic acid and syringin have no cytotoxicity to PAMs with the tested concentrations. However, the cell viability in other compounds treated cell was decreased with the higher concentrations. The cell viability rate was higher than 100% in treated cells with Baicalin, caffeic acid, chlorogenic acid, glycyrrhizic acid and tea saponin, suggested these compounds promoted the proliferation of PAMs at the concentrations used. The MNTC of each compound shown in Table 1 was used in the following anti-PRRSV assay.

**Table 1:**
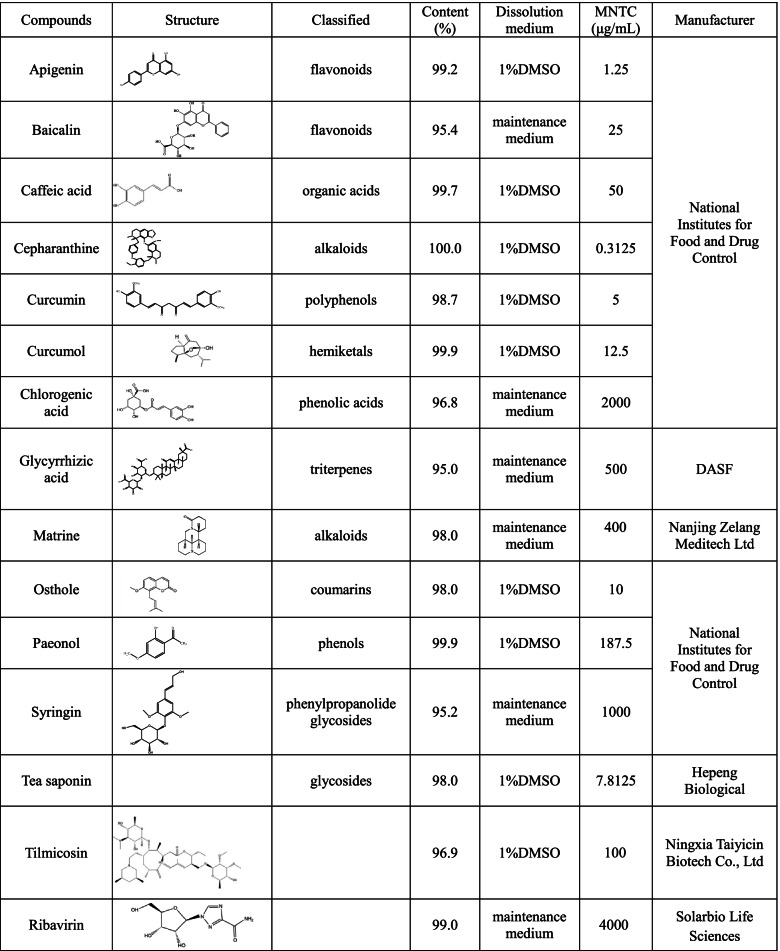
Summary of 15 compounds

QPCR and western blot were used to determine the anti-PRRSV activity of the selected compounds and the results were shown in Fig. 1B and Fig. 1C. Compared with PRRSV infected group, the PRRSV N gene mRNA expression significantly decreased in groups treated with chlorogenic acid (1 mg/ml and 2 mg/ml), curcumin (0.0125 mg/ml, 0.025 mg/ml and 0.05 mg/ml), glycyrrhizic acid (0.25 mg/ml and 0.5 mg/ml), 0.4 mg/ml matrine, tea saponin (1.95 μg/ml, 3.9 μg/ml and 7.8 μg/ml) and ribavirin (2 mg/ml and 4 mg/ml) (*p* < 0.05). However, the N gene expression increased in cells treated with 0.5 mg/ml chlorogenic acid, apigenin, baicalin, caffeic acid, cepharanthine, curcumol, paeonol, osthole and syringin (*p* < 0.05). Tilmicosin showed no significant effect on the N gene expression (*p* > 0.05). According to qPCR results mentioned above, chlorogenic acid, currcumin, glycyrrhizic acid, matrine, tea saponin, ribavirin and tilmicosin were selected to further study their effect on N protein expression. As shown in Fig. 1C, glycyrrhizic acid, matrine and tea saponin at all of tested concentrations significantly inhibited the expression of PRRSV N protein (*p* < 0.05). Chlorogenic acid (1 mg/ml and 2 mg/ml), curcumin (0.025 mg/ml and 0.05 mg/ml) and ribavirin (2 mg/ml and 4 mg/ml) significantly decreased the N protein expression, while 0.5 mg/ml chlorogenic acid, 0.0125 mg/ml curcumin and 1 mg/ml ribavirin increased the expression of N protein (*p* < 0.05). Tilmicosin at 0.05 and 0.1 mg/ml inhibited N protein expression (*p* < 0.05) and showed no effect at 0.025 mg/ml (*p* > 0.05). Based on the above results, glycyrrhizic acid (GA), matrine (MT) and tea saponin (TS) were selected for studying effect of compound combination on the PRRSV N gene expression.

### Proteomice analysis and qPCR to identify proteins regulated by compounds

**Fig. 2:**
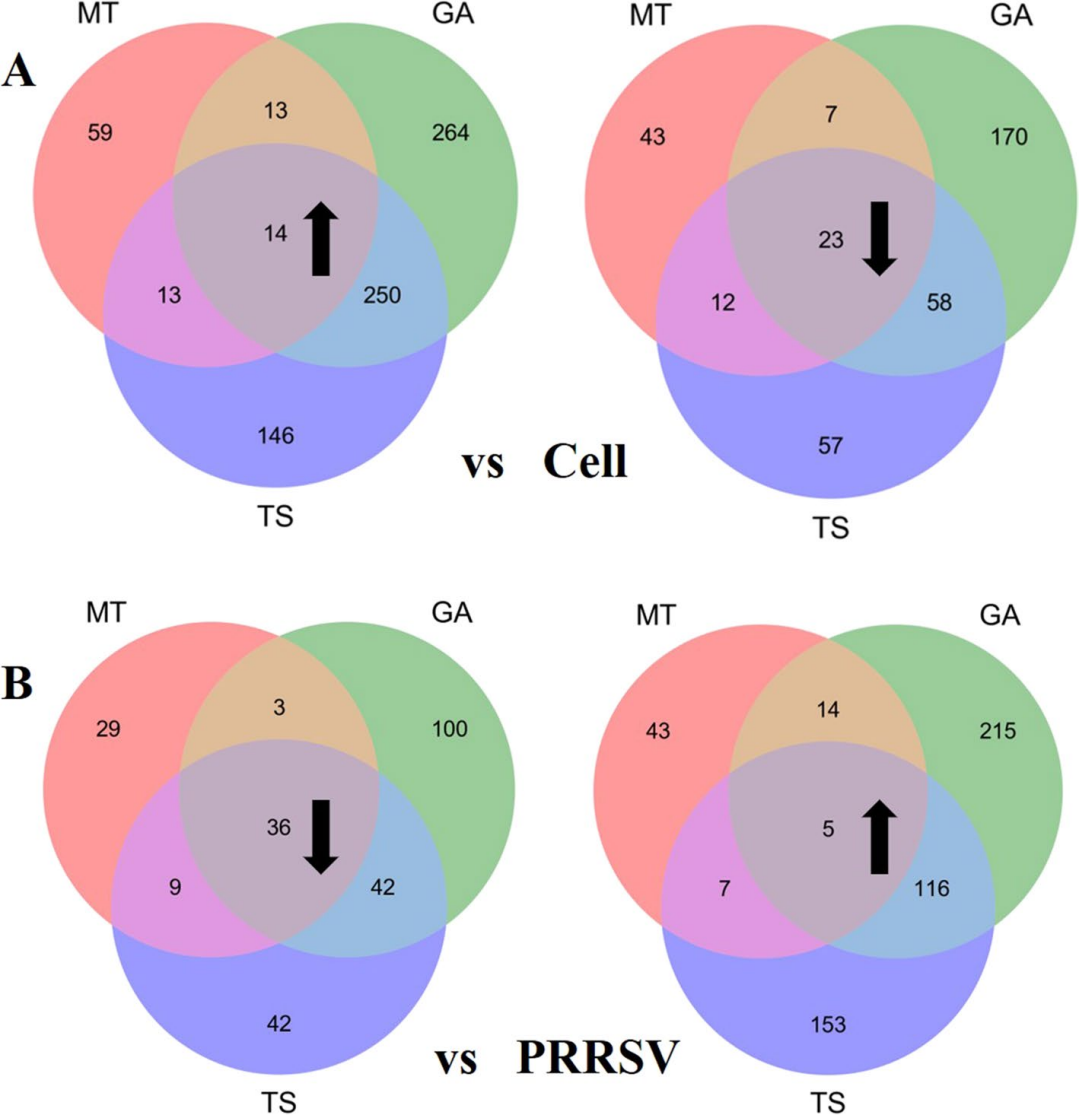
Venn diagram showing the overlap of commonly expressed proteins between three compound-treated groups and with control cell **A** and with virus alone group **B**

A TMT-based proteomic analysis was conducted to identify protein alteration in PAMs treated with PRRSV, GA, MT and TS, respectively. Expressed proteins with a fold change of > 1.20 or < 0.83 and with corrected P < 0.05 were considered to be statistically significant. The comparison of differentially expressed proteins were shown in Table 2. Compared with cell group, 101 up-regulated proteins and 62 down-regulated proteins were identified in the PRRSV infected cells. In GA treatment group, 541 proteins were up-regulated and 258 down-regulated. In MT treatment group, 99 proteins were up-regulated and 85 proteins were down-regulated; In TS treatment group, 423 proteins were up-regulated and 150 proteins were down-regulated. These differentially expressed proteins in different treatment groups compared with cell group were overlapped with 14 up-regulated and 23 down-regulated proteins (Fig. 2A). Similarly, as shown in Table 2 and Fig. 2B, compared with PRRSV group, 350 were up-regulated and 181 were down-regulated proteins among 531 expressed proteins in GA group. The total differential proteins in MT group were 146 with 69 up-regulation and 77 down-regulation. In TS group, 281 proteins were up-regulated while 129 were down-regulated among 410 identified proteins. However, only 5 were up-regulated and 36 down-regulated proteins in the overlap differential proteins among GA, MT and TS.

**Table 2:** Summary of differentially quantified proteins

Groups	Up-regulated (> 1.2)	Down-regulated (< 0.83)
V vs cell	101	62
GA vs cell	541	258
MT vs cell	99	85
TS vs cell	423	150
GA vs PRRSV	350	181
MT vs PRRSV	69	77
TS vs PRRSV	281	129

**Fig. 3:**
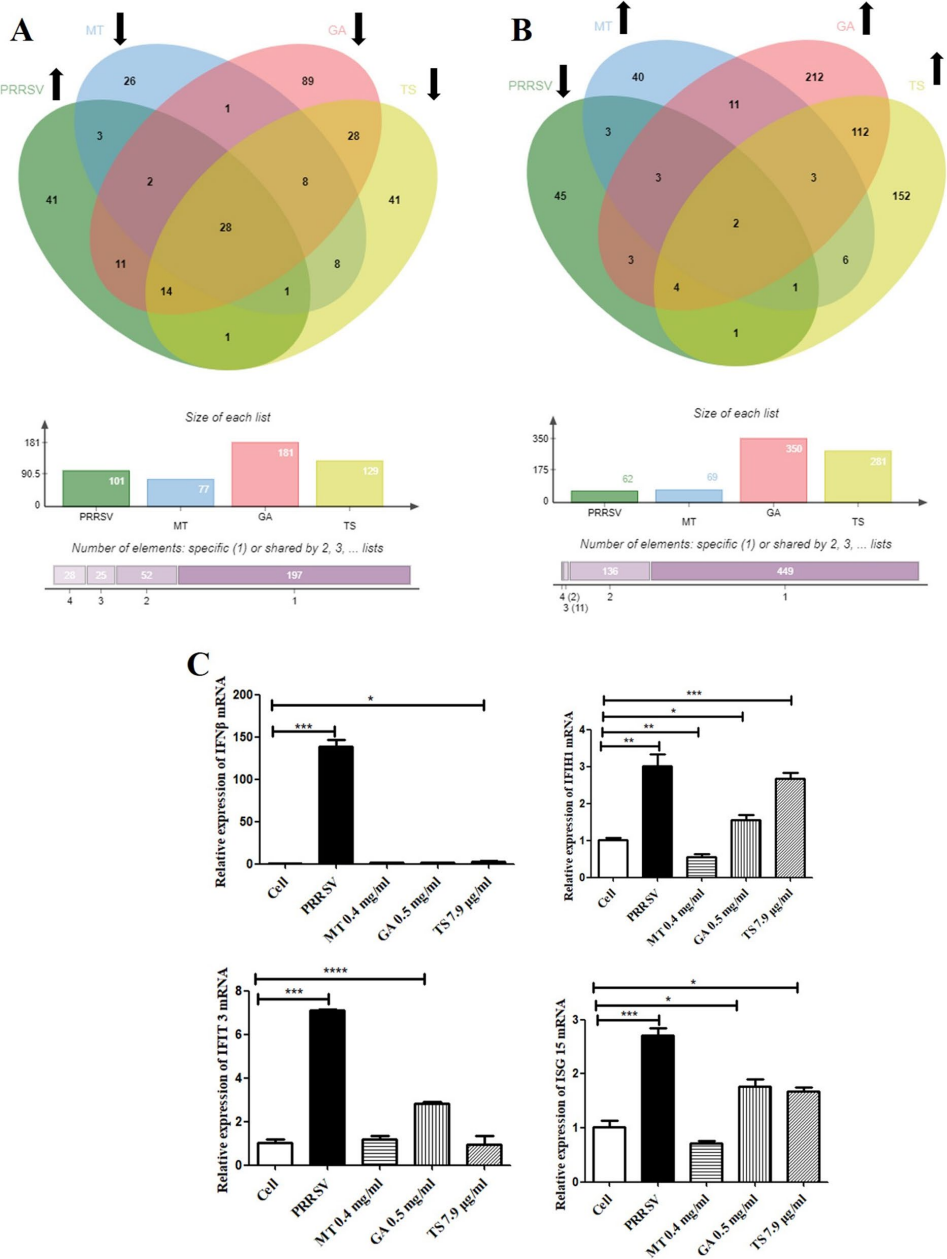
Jvenn diagrams showing the overlap of differentially expressed proteins among of up-regulated **A** or down-regulated **B** differential proteins in PRRSV group compared with cell group and the down-regulated (**A**) or up-regulated (**B**) differential proteins respectively in GA, MT and TS group compared with PRRSV group. The mRNA of top three of 28 overlapping proteins (*IFIH1*, *ISG15* and *IFIT3*) and *IFN-β* were detected by qPCR **C**. * indicated that compared with cell group, the difference was significant, *p < 0.05; **p < 0.01; ***p < 0.001

Jvenn diagrams shown the overlap of up-regulated (Fig. 3A) and down-regulated (Fig. 3B) differential proteins in PRRSV group compared with cell group as well as in GA, MT and TS group compared with PRRSV group. The results showed that GA, MT and TS decreased 28 overlap of expression proteins which was up-regulated by PRRSV infection and increased 2 of overlap expression proteins which was down-regulated by PRRSV infection. Among 28 proteins with expression overlap, the four differential expression proteins with higher expression fold change were CCL8, IFIT3, IFIH 1 and ISG15 (Table 3). IFIT3, IFIH1 and ISG15 were all related to IFN-β pathway, indicating GA, MT and TS may regulate IFN-β to exert the anti-PRRSV activity. QPCR was used to verify these results. As shown in Fig. 3C, compared with cell control, PRRSV infection significantly increased the mRNA expression of IFN-β (*p* < 0.001), IFIH1 (*p* < 0.01), IFIT3 (*p* < 0.001) and ISG15 (*p* < 0.001), suggesting that PRRSV infection activated IFN-β pathway. Compared with cell control, MT significantly decreased IFIH1 mRNA expression (*p* < 0.01), no significant effect on IFN-β, IFIT3 and ISG15 mRNA expression (*p* > 0.05). GA increased the mRNA expression of IFIH1 (*p* < 0.05), IFIT3 (*p* < 0.001) and ISG15 (*p* < 0.05), but had no effect on IFN-β expression (*p* > 0.05). Meanwhile, TS increased the expression of IFN-β (*p* < 0.05), IFIH1 (*p* < 0.001) and ISG15 (*p* < 0.05), while had no effect on IFIT3 mRNA expression (*p* > 0.05). These results suggested that GA, MT and TS affected IFN-β pathway in different degrees.

**Table 3:** 28 proteins of up-regulated in PRRSV groups and down-regulated in compounds groups

No	Accession	Protein Name	Gene Name
1	A0A4X1T9X8	C–C motif chemokine	CCL8
2	F1SCY2	Interferon-stimulated protein 60	IFIT3
3	A0A480VB50	Radical S-adenosyl methionine domain-containing protein 2 (Fragment)	N/A
4	A0A4X1W935	Uncharacterized protein	N/A
5	A0A4X1VNG5	Interferon induced with helicase C domain 1	IFIH1
6	A0A287AB58	Ubiquitin-like modifier	ISG15
7	F1SCY1	Uncharacterized protein	IFIT3
8	A0A4X1UH61	Zinc finger NFX1-type containing 1 Interferon induced protein with tetratricopeptide repeats 5	ZNFX1
9	A0A4X1UZC5	Interferon induced protein with tetratricopeptide repeats 5	N/A
10	F1SMW7	Plasminogen activator inhibitor 2	SERPINB2
11	A0A4X1UMG4	Probable ATP-dependent RNA helicase DDX58	DDX58
12	A0A480K1S7	Putative E3 ubiquitin-protein ligase HERC6 isoform 1 (Fragment)	N/A
13	A0A4X1U4J7	GB1/RHD3-type G domain-containing protein	LOC100155195
14	A0A480YJW7	E3 ubiquitin-protein ligase RNF213 isoform 3	N/A
15	A0A4X1TB89	Poly [ADP-ribose] polymerase	PARP14
16	A0A287A1I0	5'-nucleotidase, cytosolic IIIA	NT5C3A
17	A0A4X1U487	HECT and RLD domain containing E3 ubiquitin protein ligase 5	HERC5
18	A0A480N6U6	Poly ADP-ribose polymerase 12	N/A
19	A0A4X1W796	Exonuclease domain-containing protein	ISG20
20	A0A481DGC9	E3 ubiquitin-protein ligase DTX3L	N/A
21	F4ZS20	Ubiquitin-conjugating enzyme E2L 6	UBE2L6
22	A0A4X1TWZ9	Tetratricopeptide repeat and ankyrin repeat containing 1	TRANK1
23	F1RGN6	F-box protein 39	FBXO39
24	A0A4X1TD18	Poly(ADP- ribose) polymerase family member 9	PARP9
25	A0A287A342	Galectin	LGALS9
26	A0A4X1W8D9	Signal transducer and activator of transcription	STAT2
27	A0A287AP08	Myeloid cell nuclear differentiation antigen	MNDA
28	A0A4X1TGT7	Uncharacterized protein	N/A

**Fig. 4:**
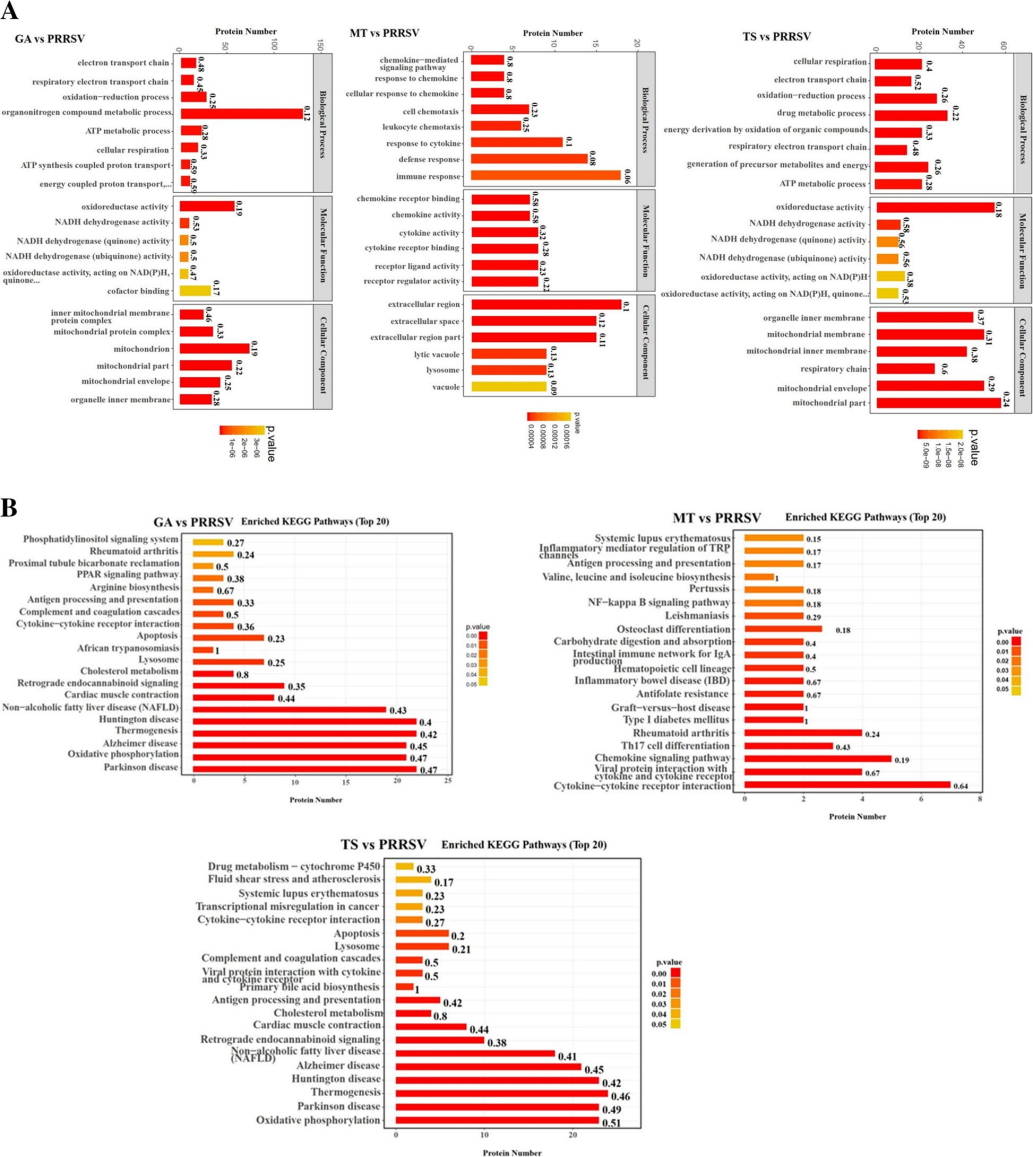
GO annotation and enrichment analysis of differentially expressed proteins of matrine, glycyrrhizic acid and tea saponin groups **A**. The enriched KEGG pathways of differentially quantified proteins of matrine, glycyrrhizic acid and tea saponin groups **B**

The differential expression of proteins was further investigated using GO and KEGG annotations. GO annotations for proteins were classified into three categories including biological process, cellular components and molecular functions. As shown in Fig. 4A, compared with PRRSV infection, the differentially expressed proteins in GA group were co-enriched in organonitrogen compound metabolic process, oxidoreductase activity and mitochondrion. The differentially expressed proteins in MT group were co-enriched in immune response, cytokine activity and extracellular region. The differentially expressed proteins in TS group were co-enriched in drug metabolic process, oxidoreductase activity and mitochondrial membrane. A KEGG pathway analysis (Fig. 4B) revealed that the differentially expressed proteins in GA group were mainly enriched in the Huntington disease, Parkinson disease, Thermogenesis and Oxidative phosphorylation. The MT group was mainly enriched in the Cytokine-cytokine receptor interaction, Chemokine signaling pathway, Rheumatoid arthritis and Viral protein interaction with cytokine and cytokine receptor. The TS group was mainly enriched in Thermogenesis, Huntington disease, Parkinson disease and Oxidative phosphorylation. Comprehensive analysis of both GO and KEGG annotations showed that the biological effects of differentially expressed proteins in both GA and TS groups were similar, while it was different with MT group. These results suggested that GA and TS may have the similar anti-PRRSV mechanism, which is different from the mechanism of MT.

### The effect of formulas on PRRSV infection

**Fig. 5:**
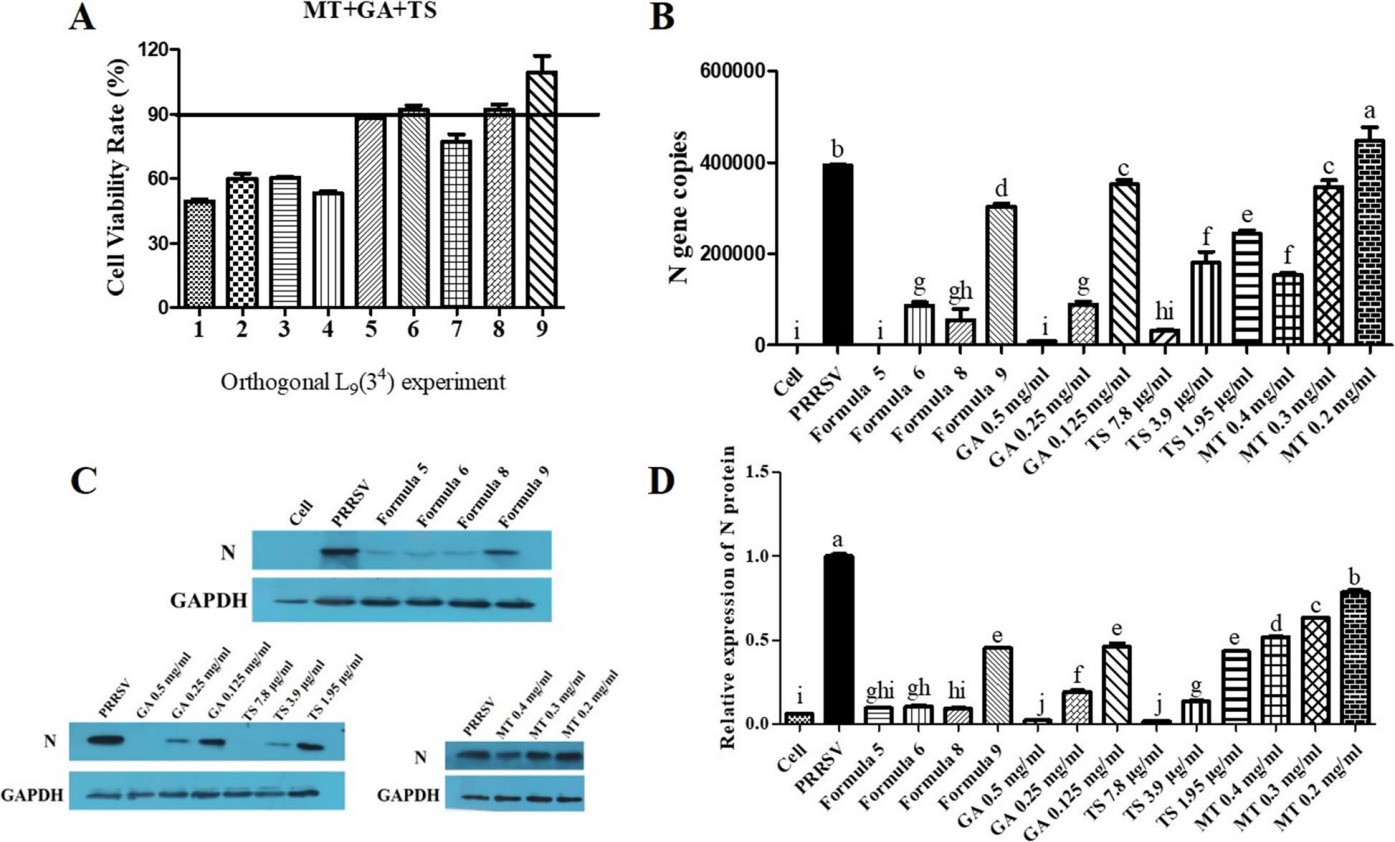
Cytotoxicity (**A)** and anti-PRRSV activity (**B**, **C** and **D**) of the component formula of Glycyrrhizic acid (GA), tea saponin (TS) and matrine (MT) on PAMs. Orthogonal L_9_ (3^4^) experiment was conducted to generate the component formulas with different concentrations of MT, GA and TS. The cytotoxicity of these nine formulas was determined by MTT method **A**. The expression of PRRSV N gene and protein were determined by qPCR **B** and western blot **C** and **D**. Cropped bolts are displayed. Different letters (a-i) showed significant difference among groups, *p* < 0.05

Nine formulas were generated by combination of compounds from MT, GA and TS at different concentration for each compound according to orthogonal L_9_(3^4^) experiment design and the cytotoxicity of these nine formulas were determined by MTT method. As showed in Fig. 5A, more than 90% of the cell viability were observed in cell treated with formulas 5, 6, 8 and 9. Therefore, formula 5 (0.25 mg/mL GA + 3.9 μg/mL TS + 0.2 mg/mL MT), formula 6 (0.25 mg/mL GA + 1.95 μg/mL TS + 0.4 mg/mL MT), formula 8 (0.125 mg/mL GA + 3.9 μg/mL TS + 0.4 mg/mL MT) and formula 9 (0.125 mg/mL GA + 1.95 μg/mL TS + 0.3 mg/mL MT) were chosen to further investigate their anti-PRRSV activity by detecting PRRSV N gene/protein using qPCR and western blot, respectively. Compared with PRRSV group, the four formulas significantly decreased both N gene (Fig. 5B) and protein (Fig. 5C and 5D) expression (*p* < 0.05). The N protein level in formula 9 treated group much higher than that in the other three-formula treated groups (*p* < 0.05). Compared with the maximum dosage used alone for GA (0.25 mg/mL), MT (0.4 mg/mL) or TS (3.9 μg/mL), formula 5, 6 and 8 showed stronger or equivalent inhibitory effect on the expression of PRRSV N gene/protein, suggested that GA, MT and TS have synergistic effect on anti-PRRSV.

**Fig. 6:**
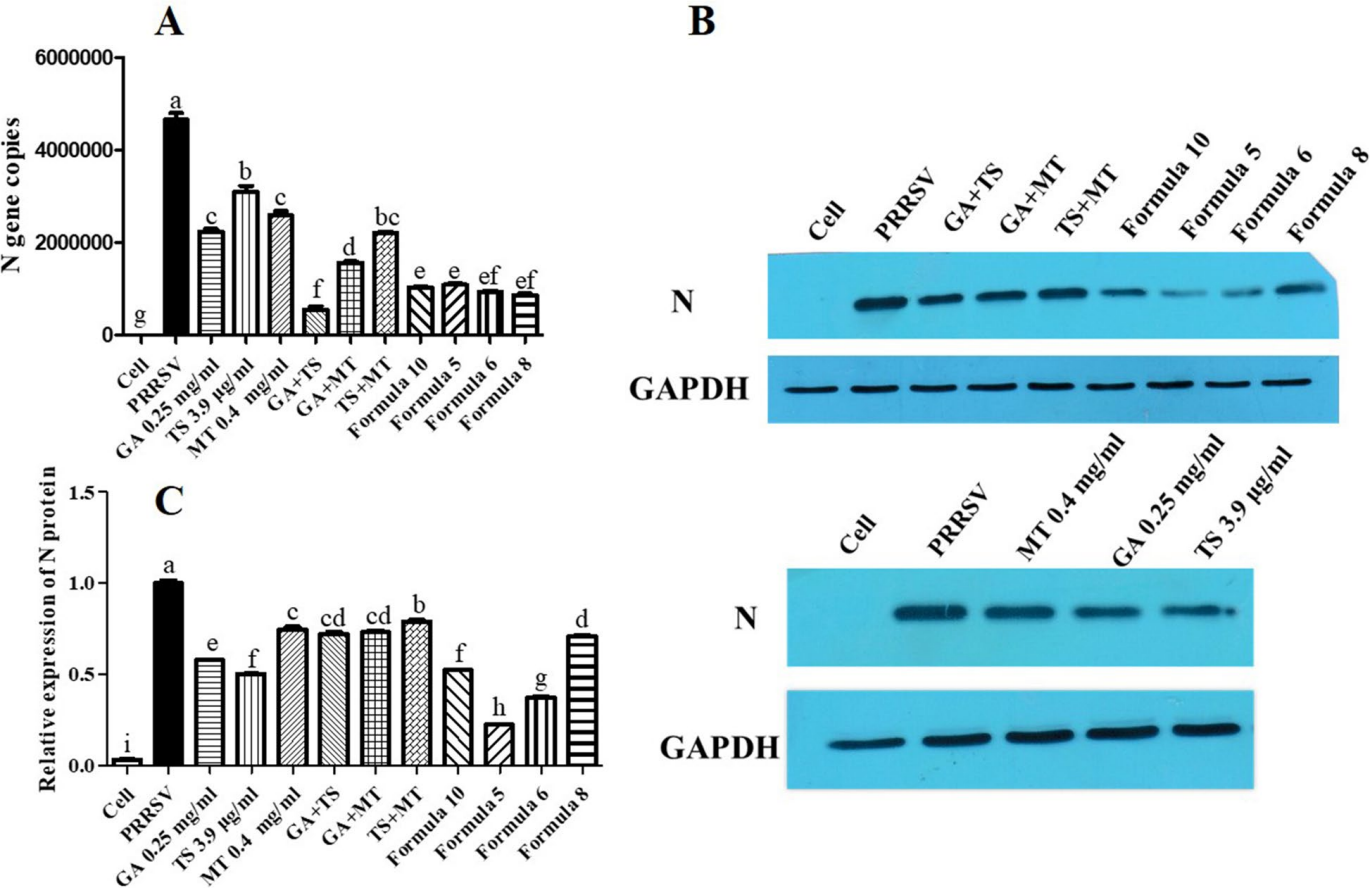
The compound concentration in the formula were further optimized and the synergistic antiviral effect of GA, TS and MT were verified by qPCR (**A)** and western blot (**B** and **C)**. Cropped bolts are displayed. Letters (a-i) showed significant difference among groups, *p* < 0.05

In order to further verify the synergistic antiviral effect of GA, MT and TS, combination of 0.25 mg/mL GA, 0.4 mg/mL MT and 3.9 μg/mL TS were used to cells infected with PRRSV. Compared with PRRSV infected group, the expression of N gene (Fig. 6A) and protein (Fig. 6B) was significantly decreased in compound alone group, pairwise or triple combination groups among GA, MT and TS (*p* < 0.05). The inhibitory effect in group treated with pairwise combination was not significantly stronger than that in compound alone. N gene/protein expression were significantly decreased both in formula 5 and 6, compared with the compound alone or pairwise combination (*p* < 0.05). Considering the cost of formula, formula 6 (0.4 mg/mL MT + 0.25 mg/mL GA + 1.95 μg/mL TS) was selected to further investigate its antiviral mechanisms.

### The formula 6 inhibited IFN-β pathway activated by PRRSV infection

**Fig. 7:**
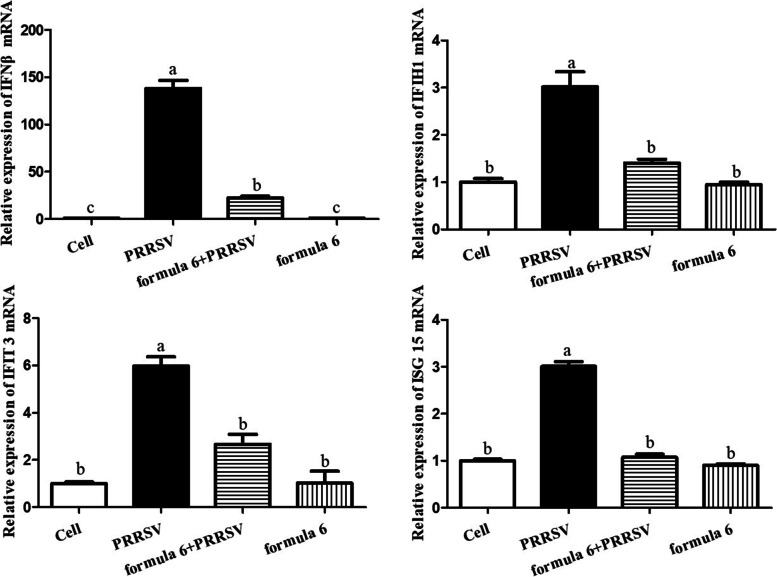
The component formula 6 inhibited the mRNA expression of *IFN-β*, *IFIH1*, *ISG15* and *IFIT3*. Different letters (**a**-**c**) showed significant difference among groups, *p* < 0.05

Formula 6 and PRRSV were simultaneously added into PAMs and co-cultured for 12 h. IFN-β, IFIH1, IFIH3 and ISG15 mRNA expression were determined by qPCR. Compared with the cell control, the expression of IFN-β and relative genes were significantly increased, while no difference was observed in group treated with formula 6, as shown in Fig. 7. In addition, the mRNA expression of IFN-β, IFIH1, IFIH3 and ISG15 in group treated with formula 6 was significantly decrease compared with PRRSV group. These results suggested that formula 6 inhibited IFN-β pathway activated by PRRSV infection.

## Discussion

The component formula derived from the Chinese medicines with clear mechanism of action and ingredients could make up the shortage of both single-ingredient drug and traditional Chinese medicines formula. The component formula of Chinese medicine is a theoretical innovation in the Chinese materia medica and beneficial to the modernization and globalization of traditional Chinese medicine. Nowadays, there are few reports on the component formula with clear mechanism of action and ingredient information. Lu et al. studied the anti-inflammatory activity of the component formula combination of phillyrin, emodin, baicalin and liquiritin [15]. A component formula from traditional Chinese medicines was built for hypercholesterolemia using virtual screening and biology network, which provided a useful tool to build a component formula from Chinese medicine [16]. In this study, the component formulas with the antiviral effect on PRRSV were studied. First, 13 compounds which possessed antiviral activities were screened in PRRSV infected PAMs. Glycyrrhizic acid, matrine and tea saponin showed anti-PRRSV activity in PAMs. Our previous study demonstrated that matrine [17], dipotassium glycyrrhetate [18] and tea seed saponins [19] inhibit PRRSV infection in Marc-145 cells. Moreover, these three compounds have broad spectrum of antiviral activity. Matrine has positive effect on human enterovirus 71[20], hepatitis B virus [21], HIV-1 [22], SARA-CoV-2 [23], PCV2 [24, 25], EMCV [26], etc. Sun et al. summarized the inhibitory effect of glycyrrhizic acid on hepatitis B virus, influenza virus, herpes virus and HIV [27]. Yu et al. demonstrated that glycyrrhizic acid has the antiviral SARS-CoV-2 activity by interacting with spike protein [28]. A few literatures reported that tea saponin inactivated human type A and B influenza viruses [29], and increased the antibody titer against Newcastle disease virus and infectious bronchitis virus to enhance the immunity [30]. In addition, Hu et al. demonstrated that saponin components could provide protection for PRRSV infected piglets and strengthen their immune system [31]. Platycodin D, one of the major bioactive triterpenoid saponins derived from Platycodon grandiflorum, has been demonstrated to inhibit PRRSV infection both in Marc-145 cells and PAMs [32]. Most of these studies mentioned just demonstrated compounds had the anti-PRRSV activity in vitro but did not explore the underlying mechanisms responsible for their antiviral activity in detail. Therefore, it is a long way to go for developing anti-PRRSV drug or drugs that could be used clinically.

Proteomic is the powerful tool to analyze differentially expressed proteins and understand the underlying pharmacological mechanisms of traditional Chinese medicine [33]. In this study, Tandem Mass Tag labeled proteomic analyses were conducted to decipher the target proteins and signaling pathways involved by glycyrrhizic acid, matrine and tea saponin to inhibit virus replication. Based on proteomics analysis combined with affinity MS, Wang et al. demonstrated that ras protein is a direct target of ginsenoside in cancer cells [34]. 163 differentially expressed proteins related to PRRSV infection were determined using proteomics, which provided the useful data for further understanding the mechanism of PRRSV infection. Meanwhile, 799, 184 and 573 of differentially expressed proteins was regulated by MT, GA and TS on PAMs, respectively, which are useful data for the subsequent interpretation of their pharmacological effects. According to the GO and KEGG enrichment analysis, GA and TS achieved their anti-PRRSV effect via almost the same pathways, while the action mechanism of MT was different from GA and TS. Based on the above results, we speculated that the compatibility of GA, MT and TS combination were based on the synergistic effect of GA and TS on the same targets while MT exerted the antiviral effect on different targets.

The optimal combination of GA, MT and TS were investigated by orthogonal design study. According to the results of cytotoxicity and antiviral assay, formula 5 (0.2 mg/mL MT + 0.25 mg/mL GA + 3.8 μg/mL TS) and 6 (0.4 mg/mL MT + 0.25 mg/mL GA + 1.95 μg/mL TS) showed much stronger anti-PRRSV activity. Compared with matrine, tea saponin possess a serious characteristic of poor water solubility, high price and stronger hemolytic activity, formula 6 (0.4 mg/mL MT + 0.25 mg/mL GA + 1.95 μg/mL TS) will be further investigated in vivo.

Innate immune response mediated by interferon (IFN) is the first line of defense against the invasion of pathogenic microorganisms. Studies have been confirmed that IFN induced cellular antiviral response is the main defense mechanism [35, 36]. The PRRSV infection inducing IFN production has been controversial because of the difference in virus strains, infection time and detection method. Overend et al. demonstrated that IFN-β expression level in PAMs was affected by PRRSV strains. When PAMs were infected with PRRSV for 12 h, ch-67 strain significantly promote IFN-β expression while CH-1 strain inhibited it [37]. In our study, the results of proteomics and qPCR validation showed that IFN-β and interferon stimulated genes (IFIH1, IFIT3 and ISG15) were up-regulated in PAMs infected with PRRSV at 12 h. It has been reported that PRRSV nonstructural protein 2 (Nsp2) antagonizes the antiviral activity of ISG15 [38], and Nsp3 induce the proteasome dependent degradation of IFITM1 upon PRRSV infection [39]. Suebsaard et al. demonstrated that rutin α-tocopherol, and l-ascorbic acid inhibit PRRSV by promoting IFN I/II and ISGs expressions [40]. The antiviral response against PRRSV of vaccine strain was induced by the secretion of extracellular ISG15 [41]. Our qPCR results showed that MT, GA and TS promoted IFN-β and interferon stimulated genes (IFIH1, IFIT3 and ISG15) expression in un-infected PAMs with varying degrees, suggesting that GA, MT and TS inhibit the PRRSV replication by targeting IFN-β pathway. The component formula of GA, MT and TS inhibited IFN-β and ISGs in PRRSV infected PAMs, but did not in un-infected PAMs. These results revealed the complicated interaction among PRRSV, IFN-β pathway and component formula.

## Conclusion

The component formula with glycyrrhizic acid, matrine and tea saponin demonstrated the anti-PRRSV activity in PAMs, indicating combination of compounds will greatly helpful to the modernization of TCM formulas. Glycyrrhizic acid and tea saponin shown the synergistic antiviral effect through regulating the same proteins in IFN-β pathway, but matrine affected the different proteins in the same pathway. Therefore, IFN-β pathway is the potential target in developing the novel antiviral formula.

## Methods

### Compounds, PRRSV and PAMs

Thirteen compounds extracted from traditional Chinese medicine were used to evaluate their anti-PRRSV activity. Ribavirin [42] and tilmicosin [43] were used in the antiviral assay as positive controls.

Details of the candidate compounds were shown in Table 1. PAMs were isolated from bronchoalveolar lavage fluid according to our previous description [44]. The strain of PRRSV used in this study was clinically isolated from one suspected PRRSV infected piglet by our lab. The identity of isolated virus was confirmed by PCR and sequences alignment analysis for PRRSV N, GP5 and NSP2 using BLAST. The similarity of nucleotide sequence of N and GP5 gene was 98.99% and 99.34%, respectively, and the amino acid sequence similarity of NSP2 was 97.52% with JXAI. The isolated virus was then propagated on Marc-145 cells and the virus titer of 10^7.79^ TCID_50_/ml was determined by the Reed-Muench method.

### Cytotoxicity assay

5 × 10^4^ PAMs per well were seeded into 96-well plate. After 2 h of incubation, the non-adherent cells were discarded and the adherent cells were continuously incubated for 24 h in 96-well plates. The screened compounds were serially diluted (twofold) to generate 6 concentration gradients with RPMI-1640 containing 2% fetal cattle serum (FCS). The cell control was just added RPMI-1640 containing 2% FCS. Four replicates of each concentration were set up. Subsequently, the medium was removed and the pre-prepared compounds were added to the corresponding wells. Post 72 h of incubation, the medium containing compounds were discarded, 20 μl MTT was added to each well and incubated for 4 h at 37 °C. The MTT was then removed and 150 μl DMSO was added to dissolve the formazan crystals. The optical density was measured using a microplate reader at 490 nm wavelength. It was calculated that the maximum non-cytotoxic concentration (MNTC) which is a compound concentration maintaining 90% cell viability.

### Anti-PRRSV screening

PAMs were seeded into 6-well plate at a density of 5 × 10^5^/ml. Three dilutions were made for each of selected compound. MNTC was used as the highest final concentration for each compound. The compound volume added to each well was equal to the volume of PRRSV at final virus titer of 10^5.79^TCID_50_/ml. Cell control group (2 ml RPMI-1640 containing 2% FCS) and PRRSV control (2 ml 10^5.79^TCID_50_/ml PRRSV) were simultaneously set up. After 12 h incubation with compound and virus, PAMs from all treatment groups were collected, RNA and protein were extracted to determine the PRRSV N gene/protein expression.

### Quantitative real-time polymerase chain reaction (qPCR)

Total RNA was extracted from PAMs using TRIzol reagent (Takara, China) according to the manufacturer’s protocols. Subsequently, the concentration of RNA was determined using a NanoDrop 1000 spectrophotometer (NanoDrop Technologies, USA). CDNA was synthesized using Prime Script™ RT Reagent kit with gDNA eraser (TaKaRa, China). QPCR was performed using SYBR Green qPCR Master Mix with high ROX from Bimake according to the applied biosystems®7500 real-time PCR system. Primer sequences and PCR product sizes were described in Table 4. A standard curve was generated using serially tenfold diluted plasmid containing N gene for each experiment.

**Table 4:** Primer sequences and the size of PCR products

Gene	Primer Sequences	PCR products (bp)
PRRSV-N	F: AGAAGCCCCATTTCCCTCTA R: CGGATCAGACGCACAGTATG	196
IFN-β	F: TGCATCCTCCAAATCGCTCT R: ATTGAGGAGTCCCAGGCAAC	139
IFIT3	F: TGCAGCCCAACAGTCTTTAG R: CTTGCAGCAGGTCTCCATC	170
IFIH1	F: CTGCTATCTCATCTCGTGTT R: TCTGCTCCTTCACCTCTG	104
ISG15	F: CGTGCAAGCTGACCATTTCT R: ATACACGGTGCACATAGGCT	100
GAPDH	F: TTGGCTACAGCAACAGGGTG	166
	R: CAGGAGATGCTCGGTGTGTT	

### Western blot analysis

PAMs from all treatment groups were collected and washed twice with PBS. Total protein was extracted with cell lysis buffer (the main composition is 20 mM Tris (pH7.5), 150 mM NaCl, 1% Triton X-100, sodium pyrophosphate, β-glycerophosphate, EDTA, Na3VO4 and leupeptin; Beyotime Biotechnology, China) supplemented with protease inhibitors according to the manufacturer’s instructions. The protein concentrations were determined using a bicinchoninic acid protein assay (BCA, Beyotime Biotechnology, China). 40 μg proteins from each sample was separated on 15% SDS-PAGE, the SDS-PAGE gel was cut according to the KD of target proteins and comparison with the marker, the cropped gel was then transferred onto a polyvinylidene fluoride (PVDF) membrane (Millipore, USA). The membrane was blocked with 5% nonfat milk for 2 h at room temperature and then incubated with anti-PRRSV N monoclonal antibody (1:2000) or anti-GAPDH antibody (1:5000; Proteintech, China) for 2 h at 37 °C. After three times washing with TBST, the membrane was incubated for 1 h with the HRP-conjugated secondary antibody at 37 °C. After another three times washing with TBST, the protein bands were detected with exposure to X-ray film using an eECL Western Blot Kit (CWbio Inc., China).

### Tandem mass tag labeled proteomic analyses

*Sample collection*: PAMs were seeded into 6-well plate at a density of 5 × 10^5^/ml. After the medium was discarded, 10^5.79^TCID_50_/ml PRRSV, 0.4 mg/ml Matrine (MT), 0.5 mg/ml Glycyrrhizic acid (GA) and 7.8 μg/ml Tea saponin (TS) was respectively added into wells in triplicates for each for 12 h incubation. Cell control group (2 ml RPMI-1640 containing 2% FCS) was simultaneously set up. Cells were washed twice with PBS, collected and frozen in liquid nitrogen for proteomic analyses. The proteomic analyses were conducted by Shanghai Applied Protein Technology including the following steps: *Protein extraction and digestion*: All cell samples were lysed with SDT buffer (4%SDS,100 mM Tris–HCl, 1 mM DTT, pH 7.6) and then boiled for 15 min. After centrifuged at 14,000 g for 40 min, the supernatant was quantified with the BCA Protein Assay Kit (Bio-Rad, USA), and purity was determined by SDS-PAGE. Protein digestion by trypsin was performed according to filter-aided sample preparation (FASP) procedure described by Matthias Mann. The concentrations of peptides were calculated according to OD280. *TMT Labeling and Peptide fractionation with high pH Reversed-Phase:* 100 μg peptide mixture of each sample was labeled using TMT reagent according to the manufacturer’s instructions (Thermo Fisher Scientific). After TMT labelling, the labeled peptides were fractionated by High pH Reversed-Phase Peptide Fractionation Kit (Thermo Scientific). Three independent biological replicates were analyzed. *Liquid chromatography/tandem mass spectrometry (LC–MS/MS) analysis*: The equal amounts of the peptides were loaded onto a reverse phase trap column (Thermo Scientific Acclaim PepMap100, 100 μm × 2 cm, nanoViper C18) connected to the C18-reversed phase analytical column (Thermo Scientific Easy Column, 10 cm long, 75 μm inner diameter, 3 μm resin) in buffer A (0.1% Formic acid) and separated with a linear gradient of buffer B (84% acetonitrile and 0.1% Formic acid) at a flow rate of 300 nl/min controlled by IntelliFlow technology. LC–MS/MS analysis was performed on a Q Exactive mass spectrometer (Thermo Scientific) in the positive ion mode. MS data was acquired using a data-dependent top10 method dynamically choosing the most abundant precursor ions from the survey scan (300–1800 m/z) for higher-energy collisional dissociation (HCD) fragmentation. Automatic gain control (AGC) target was set to 3e6, and maximum inject time to 10 ms. Dynamic exclusion duration was 40.0 s. Survey scans were acquired at a resolution of 70,000 at m/z 200 and resolution for HCD spectra was set to 17,500 at m/z 200, and isolation width was 2 m/z. Normalized collision energy was 30 eV and the underfill ratio was defined as 0.1%. MS/MS spectra were searched using MASCOT engine (Matrix Science, London, UK; version 2.2) embedded into Proteome Discoverer 1.4. *Protein identification and bioinformatic analysis:* Proteins with expression ratios higher than 1.2 or lower than 0.8 are considered as significantly up- and down- regulated. The bioinformatics analysis of significant proteins was further performed by Gene Ontology (GO) annotation and Kyoto Encyclopedia of Genes and Genomes (KEGG) database [45, 46]. The potential target proteins were selected and then validated by qPCR using the 2^−△△CT^ method. GAPDH was used for normalization.

### Orthogonal experimental design

The orthogonal experimental design of L_9_(3^4^) (Table 5) was used to explore the optimal combination formula for MT, GA and TS. Three concentrations for each compound were applied to generate nine combinations, named formula 1–9. Firstly, the cytotoxicity of these nine combinations was determined by MTT method described as cytotoxicity assay. The combination groups with more than 90% of cell viability were selected and their anti-PRRSV activity were determined through N gene/protein expression by qPCR and western blot, respectively.

**Table 5:** The compound concentration in orthogonal experiment L_9_(3^4^)

Formula No	Glycyrrhizic acid (mg/ml)	Tea saponin (μg/ml)	Matrine (mg/ml)
1	0.500	7.80	0.400
2	0.500	3.90	0.300
3	0.500	1.95	0.200
4	0.250	7.80	0.300
5	0.250	3.90	0.200
6	0.250	1.95	0.400
7	0.125	7.80	0.200
8	0.125	3.90	0.400
9	0.125	1.95	0.300

### Optimization of the component combination

The maximum concentration of each compound (MT, GA and TS) in the selected formulas with anti-PRRSV effect alone and with respective combination in seven groups named as MT 0.4 mg/ml, GA 0.25 mg/ml, TS 3.9 μg/ml, GA + TS (0.25 mg/ml + 3.9 μg/ml), GA + MT (0.25 mg/ml + 0.4 mg/ml), TS + MT (3.9 μg/ml + 0.4 mg/ml), formula 10 (GA 0.25 mg/ml + TS 3.9 μg/ml + MT 0.4 mg/ml). Each group and equal amounts of PRRSV were simultaneously added to PAM, and the final titer of PPRSV was 10^5.79^TCID_50_/ml. Cell control group, PRRSV group and three selected combination groups (formula 5, 6 and 8) were both set up. After 12 h of incubation, QPCR and western blot were used to determine PRRSV N gene/protein expression to assess the optimized component ratio.

### Determination of IFN-β and ISGs expression by qPCR

The optimal component formulas and PRRSV were added to PAMs and incubated for 12 h. Cell control, PRRSV and component formula alone groups were simultaneously set up. The total RNA of each group was extracted and the mRNA expression of IFN-β, IFIH1, IFIT3 and ISG15 were detected by qPCR.

### Statistical analysis

All the data were expressed as mean ± standard errors of the mean (SEM). The statistical analysis of orthogonal experimental design and optimization of the component ratio were performed using SPSS version 21.0 software (SPSS, Chicago, IL, USA) and a one-way analysis of variance (ANOVA) followed by Duncan was used to analyze the results. Letters was used to indicate significant difference among groups, *p* < 0.05. The other’ dada were analyzed by one-way ANONA (more than two groups) or *t*-test (two groups) implemented in GraphPad Prism version 5 (GraphPad software, San Diego, CA). Histograms were all obtained from GraphPad Prism. * indicated *p* < 0.05, ** indicated *p* < 0.01, *** indicated *p* < 0.001.

## Supplementary Information

**Additional file1:** **Supplementary Figure S1.** The original blot images with specific protein bands used in this study. (A) The original blots used in Figure 1C. (B) The original blots used in Figure 5C. (C) The original blots used in Figure 6B.

## Data Availability

The data supporting the conclusions of this article are included within the article. The datasets generated and analyzed during the current study are available from the corresponding author on reasonable request.

## Abbreviations

BCA: Bicinchoninic acid
FCS: Fetal cattle serum
GA: Glycyrrhizic acid
GO: Gene Ontology
HBV: Hepatitis B virus
HSV-1: Herpes simplex virus type 1
H1N1: Influenza virus A
IFN: Interferon
KEGG: Kyoto Encyclopedia of Genes and Genomes
MNTC: Maximum non-cytotoxic concentration
MT: Matrine
PAMs: Porcine alveolar macrophages
PRRS: Porcine Reproductive and Respiratory Syndrome
qPCR: Quantitative polymerase chain reaction
SEM: Standard errors of the mean
TCM: Traditional Chinese medicine
TMT: Tandem Mass Tag
TS: Tea saponin
