# Supplementary material for: A novel strategy for optimal component formula of anti-PRRSV from natural compounds using tandem mass tag labeled proteomic analyses
Source: BMC Vet Res. 2022 May 14;18:179. doi: 10.1186/s12917-022-03184-w (PMC9106989; doi:10.1186/s12917-022-03184-w)

**Supplementary Figure S1.** The original blot images with specific protein bands used in this study. (A) The original blots used in Figure 1C. (B) The original blots used in Figure 5C. (C) The original blots used in Figure 6B.

**.**

**A**


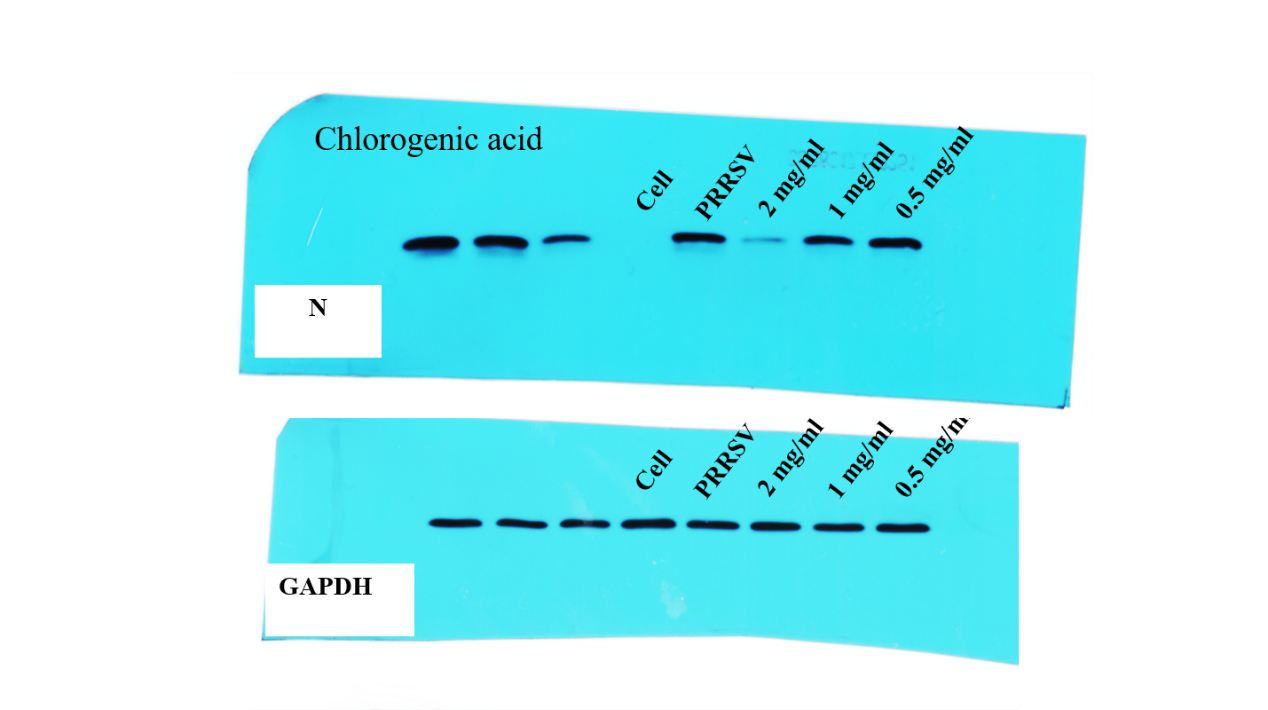


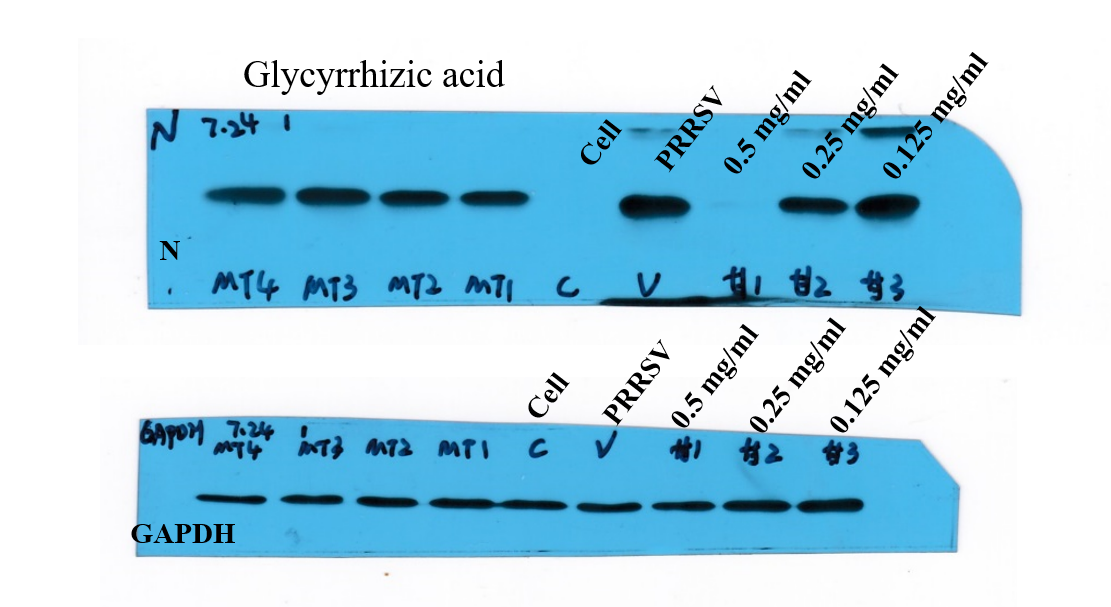


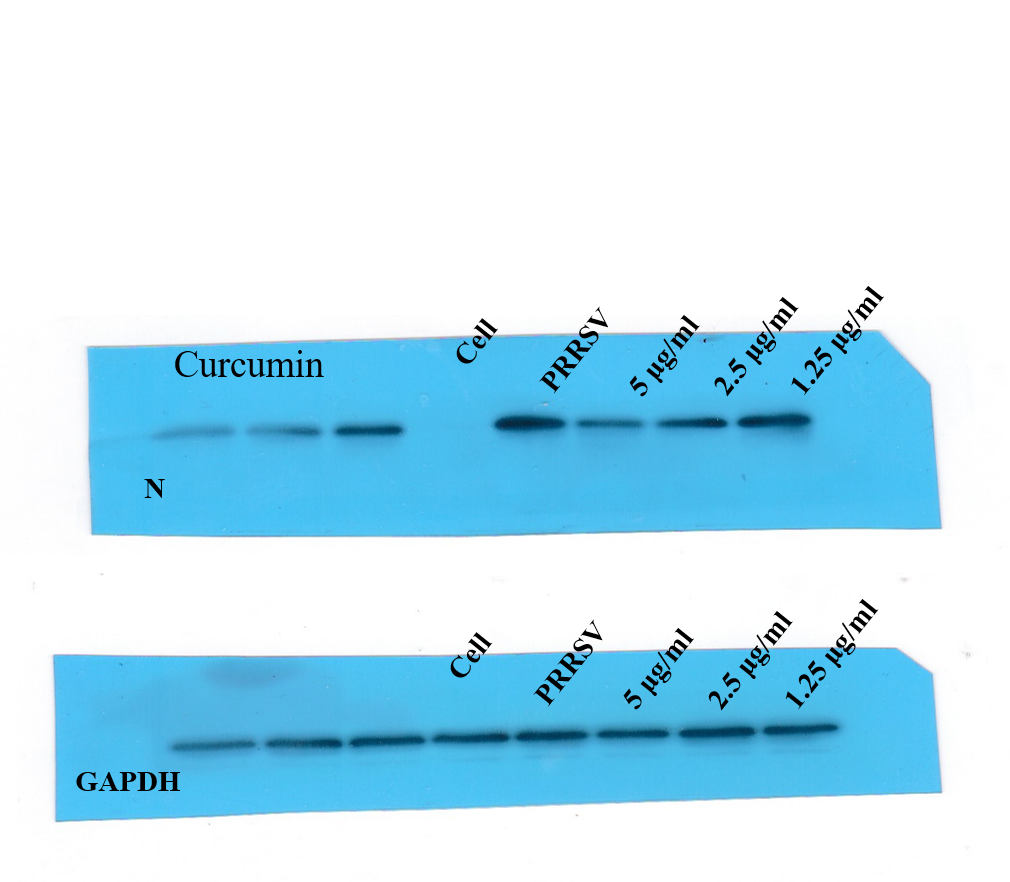


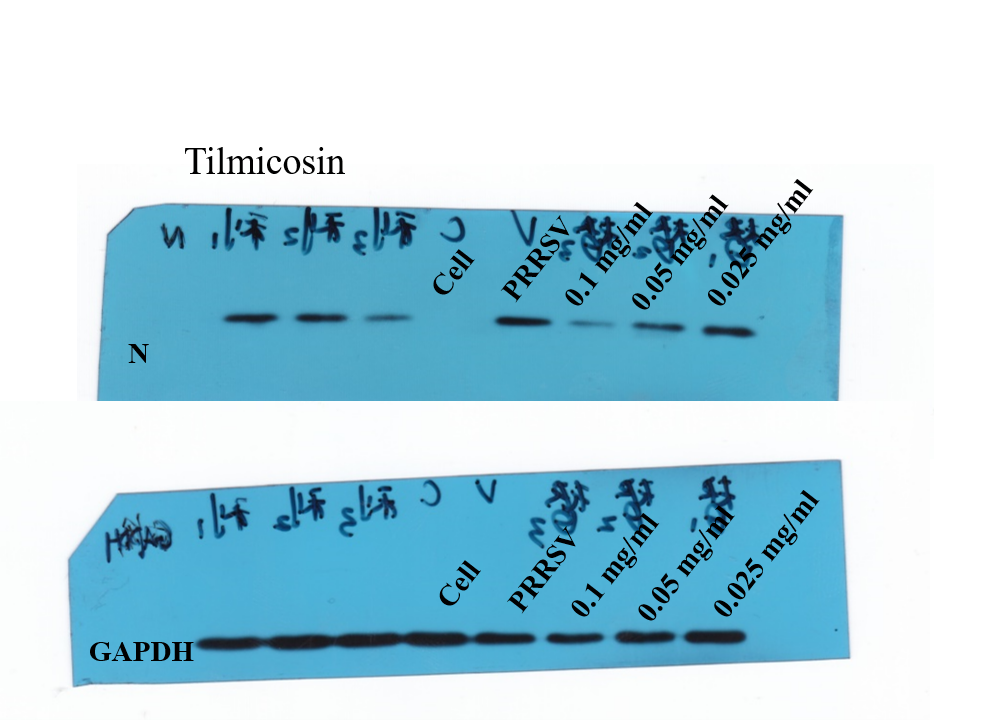


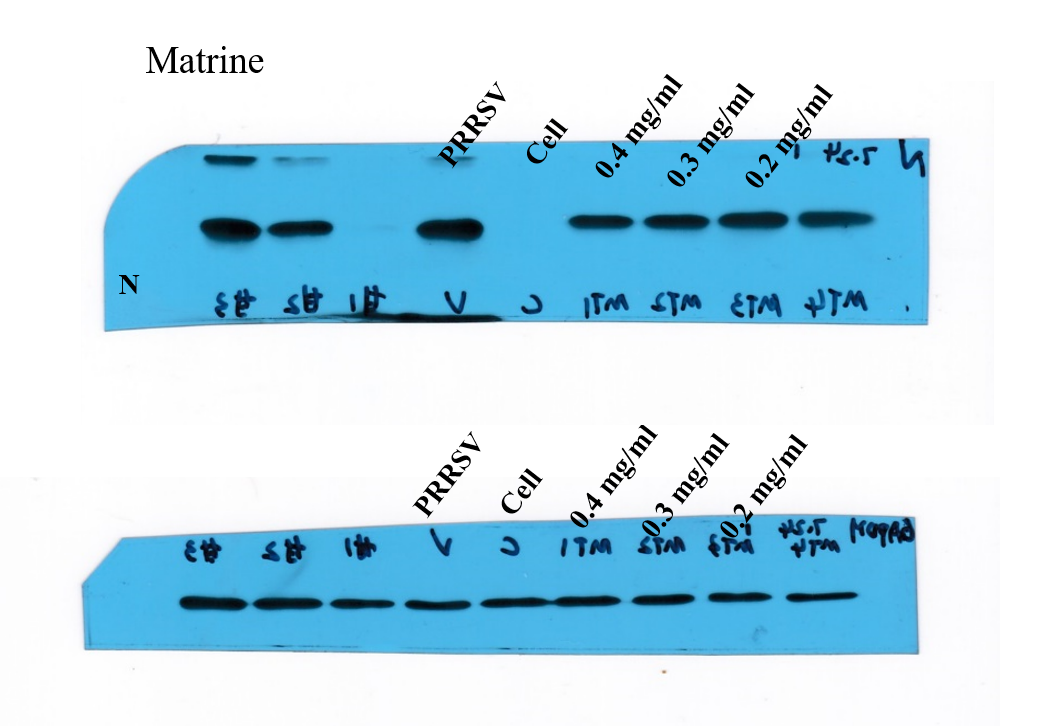


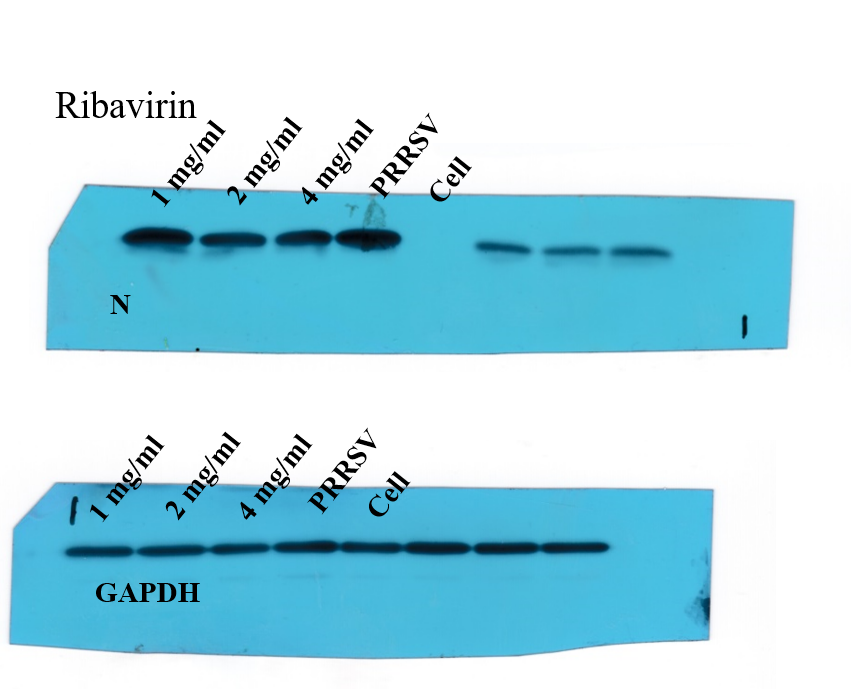


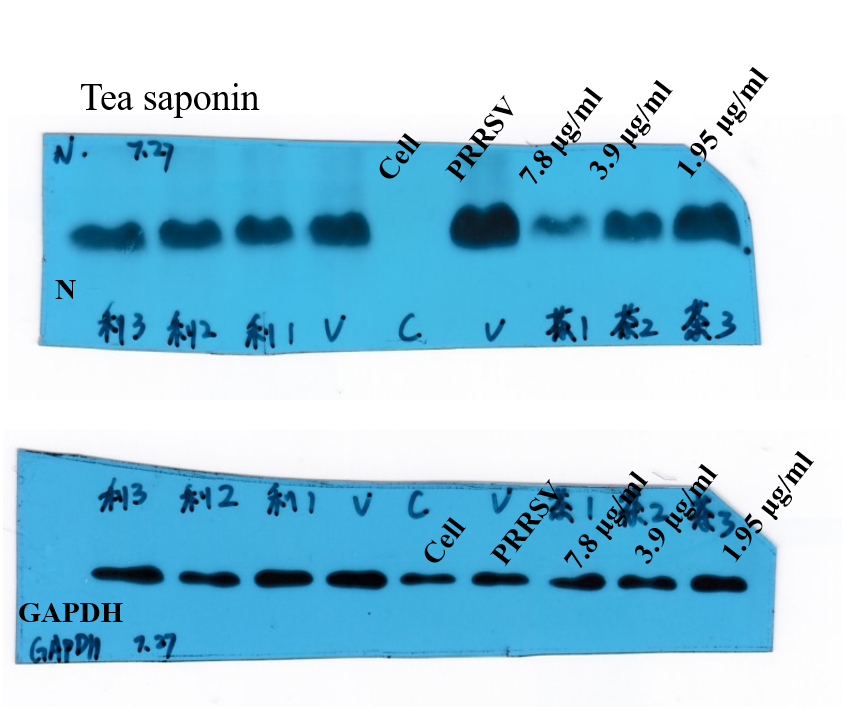


**B**


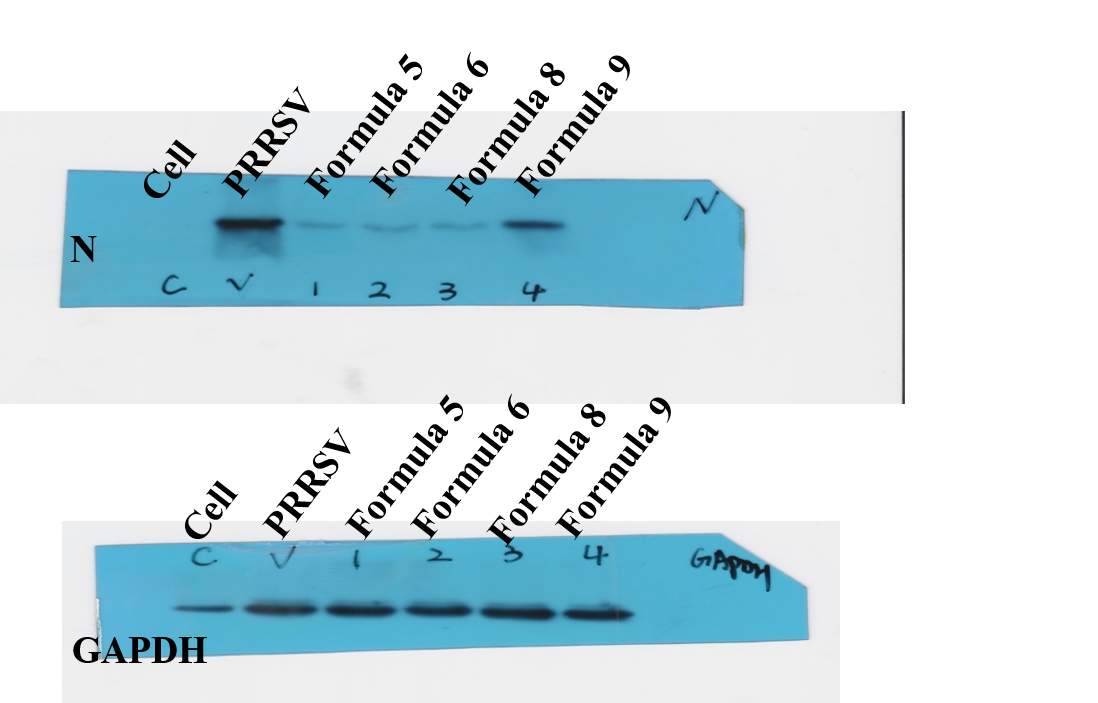


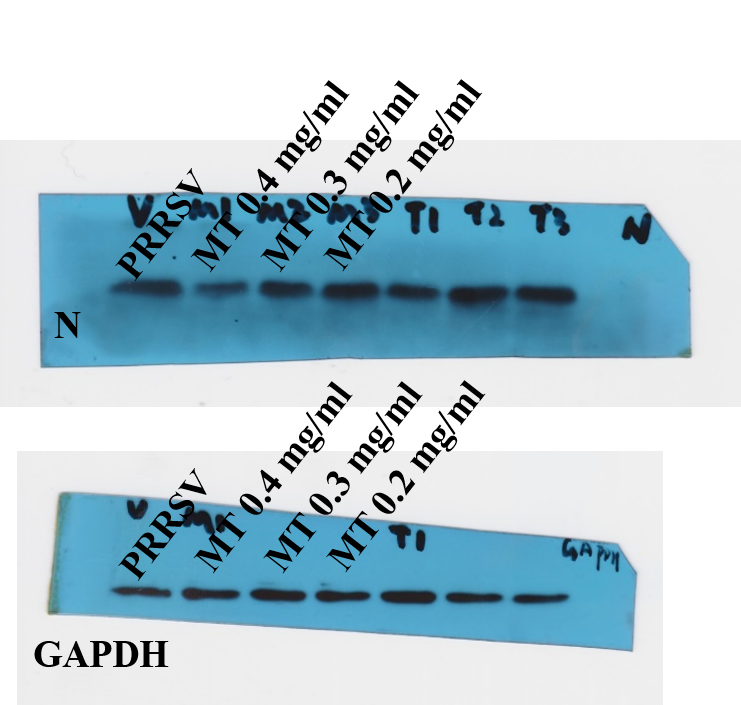


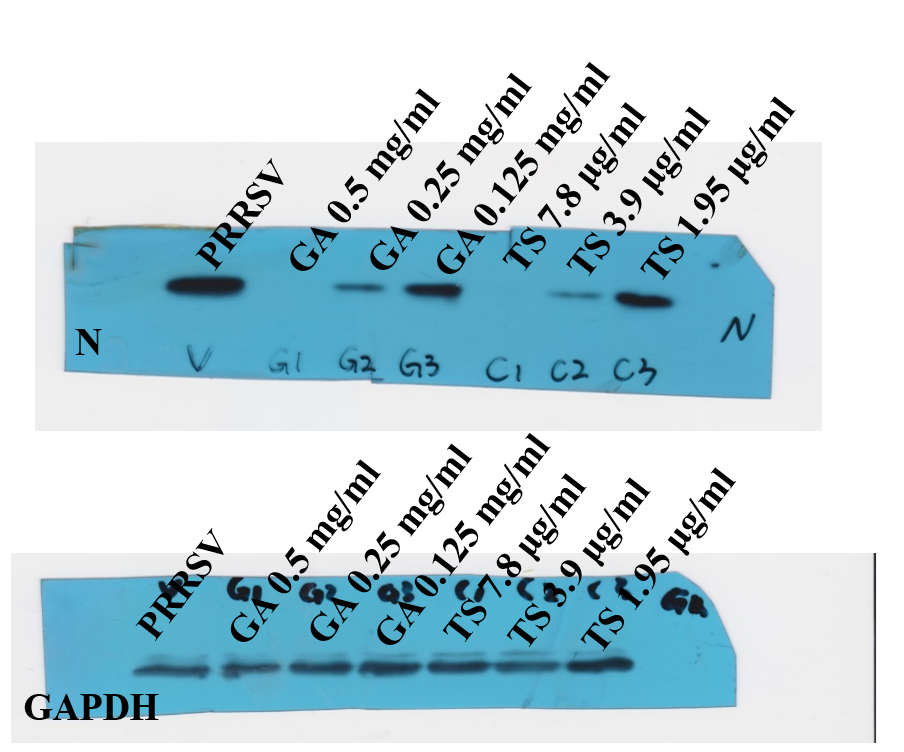


**C**


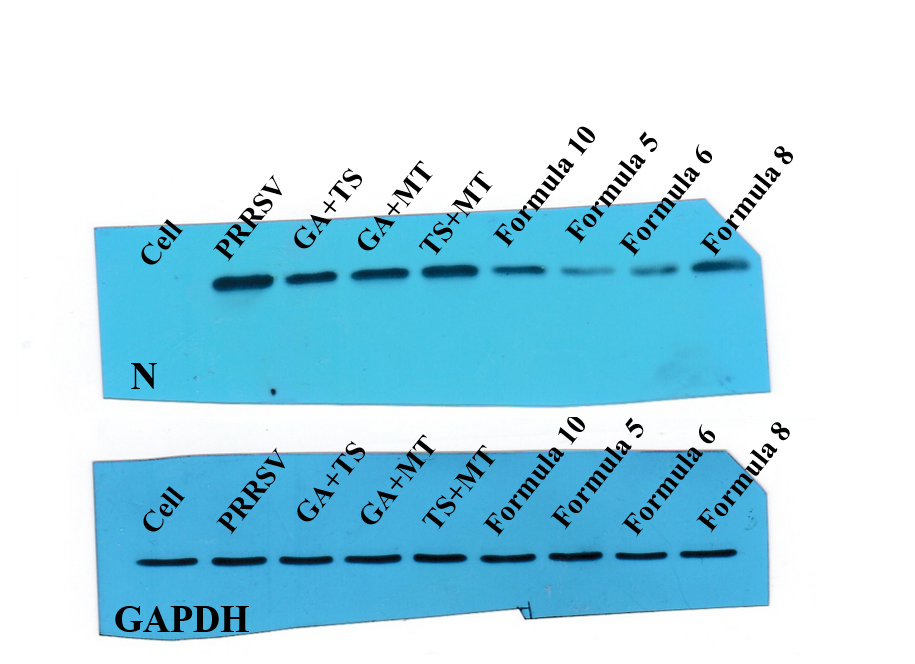

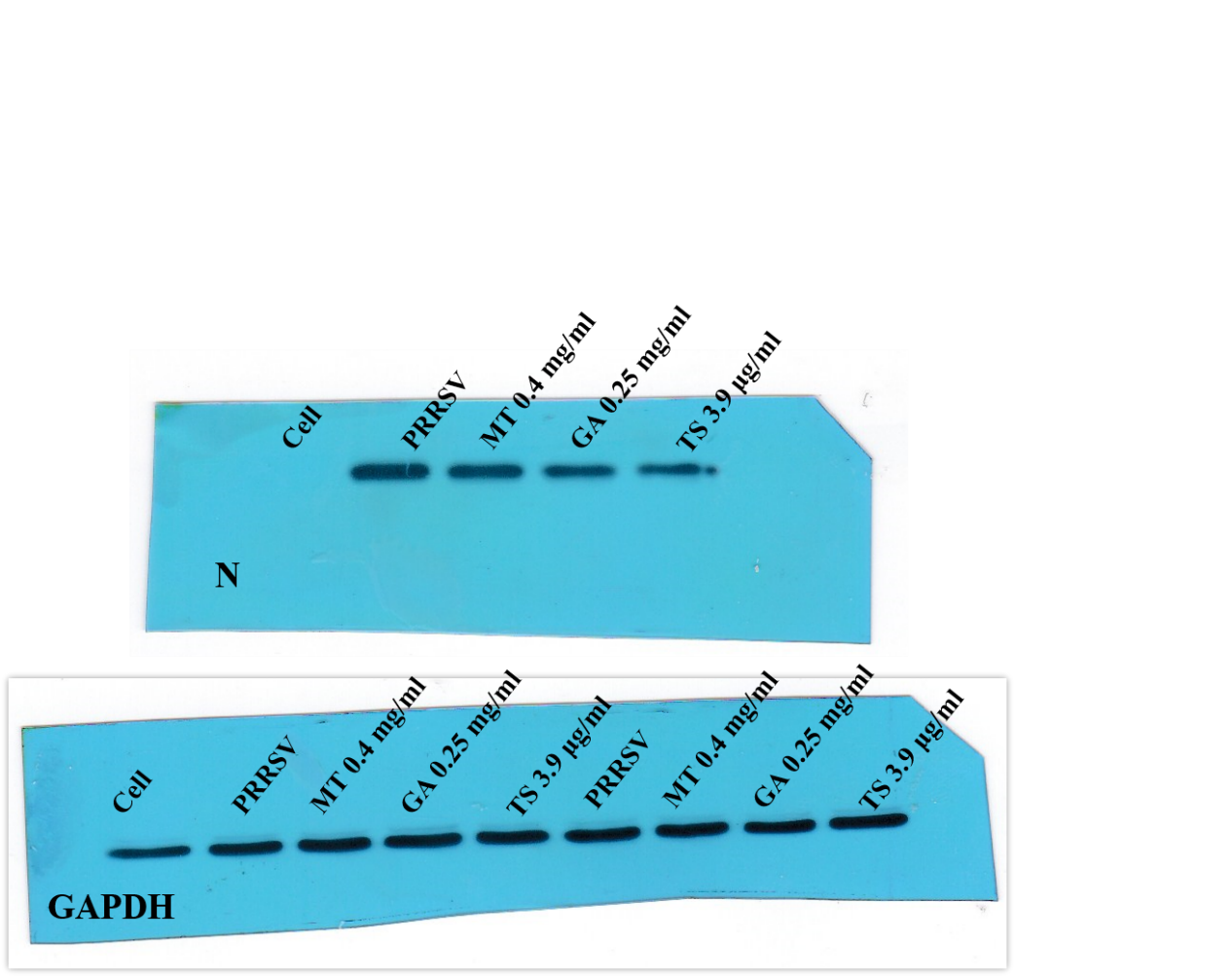

Supplement: Supplementary file 1 — Additional file1: Supplementary Figure S1. The original blot images with specific protein bands used in this study. (A) The original blots used in Figure 1C. (B) The original blots used in Figure 5C. (C) The original blots used in Figure 6B. [file 12917_2022_3184_MOESM1_ESM.docx]
